# ZMIX: estimating ancestry proportions using GWAS association Z-scores

**DOI:** 10.1093/bioadv/vbae128

**Published:** 2024-08-29

**Authors:** Trent Dennis, Donghyung Lee

**Affiliations:** Department of Statistics, Miami University, Oxford, OH 45056, United States; Winton Hill Business Center, P&G, Cincinnati, OH 45232, United States; Department of Statistics, Miami University, Oxford, OH 45056, United States

## Abstract

**Motivation:**

With larger and more diverse studies becoming the standard in genome-wide association studies (GWAS), accurate estimation of ancestral proportions is increasingly important for summary-statistics-based methods such as those for imputing association summary statistics, adjusting allele frequencies (AFs) for ancestry, and prioritizing disease candidate variants or genes. Existing methods for estimating ancestral proportions in GWAS rely on the availability of study reference AFs, which are often inaccessible in current GWAS due to privacy concerns.

**Results:**

In this study, we propose ZMIX (Z-score-based estimation of ethnic MIXing proportions), a novel method for estimating ethnic mixing proportions in GWAS using only association Z-scores, and we compare its performance to existing reference AF-based methods in both real-world and simulated GWAS settings. We found that ZMIX offered comparable results to the reference AF-based methods in simulation and real-world studies. When applied to summary-statistics imputation, all three methods produced high-quality imputations with almost identical results.

**Availability and implementation:**

https://github.com/statsleelab/gauss.

## 1 Introduction

For many years, Genome-Wide Association Studies (GWAS) have involved ethnically homogeneous populations, typically focusing on one or two populations such as Europeans or Asians. In 2009, Need and Goldstein (2009) reported that 96% of all subjects in GWAS were of European ancestry, 3% were of Asian ancestry, and all other populations combined constituted less than 1%. Based on an analysis by Popejoy and Fullerton of the GWAS catalog data from 2016 (Popejoy and Fullerton 2016), there was a noticeable shift in ethnic representation: 81% of all GWAS participants came from European descent, 14% came from Asian descent, 3% came from African descent, 1% came from Mixed ancestry, and all other populations made up less than 1%. This trend is encouraging, as the representation of non-European populations in GWAS increased by about 20% over those 7 years. The inclusion of diverse ethnic groups is important since GWAS insights are most applicable to the represented populations.

As the ethnic landscape of GWAS diversifies, the precise estimation of ethnic/ancestry proportions becomes increasingly critical. These estimates represent the percentage of subjects from each distinct ethnic group within a study. Studies with diverse and admixed populations can often face challenges related to population stratification unless the heterogeneity in study population is accounted for. (Zhang and Stram 2014). Population stratification can occur in heavily admixed studies when there are large enough differences in allele frequencies (AFs) between case and control groups, which are caused by systematic differences in ancestry as opposed to a true association of genotype and phenotype (Freedman *et al.* 2004). Thus when stratification is present and not accounted for, the number of spurious associations increases and the Type I error rate is inflated as a result. Many methods developed for multi-ethnic GWAS and meta-analyses now incorporate additional steps to address such heterogeneity. For example, Eigenstrat (Price *et al.* 2006) is a method used to adjust for population stratification in case-control GWAS studies. PCA is performed on the genotype data, from which a few components explaining the most variance are selected and considered axes of genetic variation. These components are then used to make ancestry adjustments for both genotypes and phenotypes, effectively controlling the Type I error rate in GWAS with heavily admixed populations. Adjustments for ethnic/ancestral heterogeneity in GWAS can also be performed using ancestry proportions, highlighting the need for accurate estimation of such weights. DISTMIX (Lee *et al.* 2015) estimates ancestry proportions and leverages them to accurately infer the ancestry-informed linkage disequilibrium (LD) matrix, ensuring control over the Type I error rate during imputation of summary statistics. Similarly, Summix (Arriaga-MacKenzie *et al.* 2021) estimates these proportions and uses them to compute ancestry-adjusted AFs. Both DISTMIX and Summix require the AF of each single nucleotide polymorphism (SNP) in a study to estimate ethnic proportions accurately. However, a privacy concern arose in 2008 when Homer *et al.* (2008) demonstrated that with access to an individual’s genotype information, it is possible to determine their presence in a mixture of DNA samples even when only minor allele frequencies (MAFs) are known. Following the publication of Homer et al.’s paper (Homer *et al.* 2008), many funding agencies hosting GWAS data restricted access to study AFs in response to privacy concerns (Homer *et al.* 2008, Uhlerop *et al.* 2013, Lee *et al.* 2015), highlighting the need for a method that does not rely on study AFs. To overcome this issue, we previously proposed DISTMIX2 (Chatzinakos *et al.* 2021), a method that estimates ethnic proportions solely using association Z-scores. While DISTMIX2 showcased the potential of the method, it faced challenges that needed to be resolved for its broader, user-friendly application. One of these problems was the lack of a robust optimization algorithm, such as linear/quadratic programming, which resulted in low accuracy. Additionally, DISTMIX2’s ancestry estimation procedure is integrated within DISTMIX2’s imputation procedure, making it unable to be used independently.

To address these challenges, we developed ZMIX (Z-score-based estimation of ethnic MIXing proportions). This method employs only association Z-score to estimate ancestry proportions in multi-ethnic GWAS, meta-analyses of multi-ethnic GWAS or multi-ethnic whole genome sequencing studies. Three key improvements in ZMIX over DISTMIX2 are (i) implementing a standalone function for ancestry estimation, (ii) employing a better SNP selection procedure, and (iii) incorporating quadratic programming to boost accuracy. The primary objective of this paper is to evaluate the performance of ZMIX by comparing it to three established methods, DISTMIX and Summix, both of which rely on SNP AFs and DISTMIX2, which utilizes an earlier Z-score-based ancestry approach. We first conducted extensive simulation studies to compare the performance of these methods under various ancestral mixture scenarios. Additionally, we benchmarked the performance of ZMIX and two allele-frequency-based methods using three real-world GWAS studies from the Psychiatric Genetics Consortium (PGC): The 2021 Bipolar Disorder (BIP) study (Mullins *et al.* 2021), the 2019 Attention-Deficit/Hyperactivity Disorder (ADHD) study (Demontis *et al.* 2019), and the 2022 Schizophrenia (SCZ) study (Trubetskoy *et al.* 2022). Given the importance of accurate mixing proportion estimates for summary-statistic based imputation procedures, we input the estimated proportions from each method into the DISTMIX imputation process and evaluated the accuracy of the imputations.

## 2 Methods

### 2.1 Reference panel

In this study, we utilized the 33 000 Genomes (33KG) reference panel (Chatzinakos *et al.* 2021), comprising 22 691 subjects from the Haplotype Reference Consortium (HRC) and 10 262 from CONVERGE (McCarthy *et al.* 2016, Cai *et al.* 2017). This panel represents diverse ethnicities, including 20 281 Europeans, 10 800 East Asians, 522 South Asians, 817 Africans, and 533 Native Americans. The HRC integrates data from 20 studies, primarily European but also drawing a broader diversity from the 26 populations in the 1000 Genomes Project phase 3 (Byrska-Bishop *et al.* 2022). CONVERGE subjects, primarily from various Chinese regions, are categorized under the overarching Asian (ASN) super population. An additional European grouping was curated from the HRC’s Orkney Island subjects (ORK). In total, 33KG features 29 populations across 5 super populations. Detailed information and abbreviations for these populations are available in Supplementary Table S1.

### 2.2 Ancestry proportion estimation methods

#### 2.2.1 DISTMIX

Originally, DISTMIX (Lee *et al.* 2015) was developed to impute association summary statistics of unmeasured SNPs within multi-ethnic GWAS. DISTMIX first infers ancestry proportions utilizing AF information, then employs these estimates to compute ancestry-informed LD. The estimated LD is subsequently utilized in the DIST (Direct Imputation of summary STatistics) procedure (Lee *et al.* 2013) for imputing association Z-scores of unmeasured SNPs. While DISTMIX is capable of both estimating ancestry proportions and conducting Z-score imputation, our primary focus here is on its procedure for estimating ancestry proportions.

DISTMIX assumes that the reference panel is composed of *K* ethnic populations, and that an individual’s genotypes from the study are a mixture of these *K* ethnic groups, each associated with ethnic weights $W={\left[w_{k}\right]}_{K\times 1}$. We denote the vector of study SNP reference allele frequencies (RAFs) as *S*. The vector of RAFs for the kth ethnicity in the reference panel is denoted $P_{k}$, and the aggregate RAF matrix for the reference population’s ethnicities is denoted $P={\left[P_{k}\right]}_{K\times 1}$. Ancestry proportions (ethnic weights) are then estimated through the following relationship:


$$\text{Cov}(S,P)=\text{Cov}\left(\sum_{k=1}^{K}w_{k}P_{k},P\right)=\text{Cov}(PW,P)=\text{Cov}(P)\cdot W.$$

Consequently, the ancestry proportions are computed as,


$$\hat{W}=\text{Cov}{\left(P\right)}^{-1}\cdot \;\text{Cov}(S,P),$$

where $\sum_{k=1}^{K}w_{k}=1$ and $0\leq w_{k}\leq 1$.

To manage the strong LD among SNPs, which can affect the accuracy of correlation calculations using all SNPs in a genome, GWAS SNPs are divided into 1000 distinct sets. For example, one set might contain every 1000th SNP, like the 1st, 1001st, 2001st, etc., ensuring significant distances between SNPs in each set and thus making them quasi-independent. This strategy enhances the reliability of estimated correlations. The weights $\hat{W}$, computed for each of the 1000 quasi-independent SNP sets, are then averaged to determine the final weight.

We implemented the ancestry proportion estimation component of DISTMIX as the “afmix()” function, integrated into the GAUSS R package (Lee and Bacanu 2024). This package is accessible at https://github.com/statsleelab/gauss. A comprehensive explanation and examples of how to use the “afmix()” function is available at https://statsleelab.github.io/gauss/articles/afmix_example.html.

#### 2.2.2 Summix

Recent studies (Guo *et al.* 2018, Hendricks *et al.* 2018, Arriaga-MacKenzie *et al.* 2021) have shown that AFs from public databases can be used as external proxy controls in case-control studies. However, these AFs often originate from studies involving admixed populations. Therefore, using such AFs without adjusting for ancestry may lead to spurious associations. To address this issue, Summix has been proposed (Arriaga-MacKenzie *et al.* 2021). Summix estimates ethnic weights (ancestry proportions) by utilizing RAF information from both the study and reference panel. The method then calculates ancestry-adjusted-AFs using the estimated weights.

Summix (Arriaga-MacKenzie *et al.* 2021) employs quadratic programming to estimate ethnic proportions. It considers *K* ethnic populations, each associated with specific mixing proportions $w_{k}$. The method estimates each $w_{k}$ by minimizing the least-squares difference between the observed AFs vector for each SNP, $AF_{\text{observed}}$, and the AFs derived from a mixture of *K* reference panel ethnic populations, $AF_{\text{ref},k}$. Therefore, the solutions for $w_{k}$ are obtained by minimizing the objective function:


$$f(w)={\left(AF_{\text{observed}}-\sum_{k=1}^{K}\left(w_{k}\cdot AF_{\text{ref},k}\right)\right)}^{2},$$

subject to the constraints: $w_{k}\geq 0,k=1,2,\ldots ,K$ and $\sum_{k=1}^{K}w_{k}=1$.

Arriaga-MacKenzie *et al.* (2021) used sequential quadratic programming to solve the equation given the constraints.

The ancestry proportion estimation algorithm of Summix is implemented within the “summix()” function in the Summix R package. This Bioconductor package is publicly available at: Https://www.bioconductor.org/packages/release/bioc/html/Summix.html.

### 2.3 ZMIX: Z-score based ancestry proportion estimation

The two methods previously discussed rely on the availability of AFs from a study, which may not always be accessible due to privacy concerns (Uhlerop *et al.* 2013). To overcome this limitation, our group has recently refined DISTMIX into a new version, now called DISTMIX2 by introducing a new procedure that estimates ancestry proportions using only GWAS association Z-scores, by passing the need for RAF (Chatzinakos *et al.* 2021).

Similar to DISTMIX, DISTMIX2 also assumes that the genotype of study cohort is a mixture of random genotypes from the *K* ethnic groups in the reference panel, with mixing proportions ${\left[w_{k}\right]}_{K\times 1}$. In this context, $G_{\mathrm{ijk}}$ is defined to represent the genotype value for the ith SNP of the jth subject, who belongs to the kth ethnic group. Additionally, let $p_{ik}$ denote the RAF of the corresponding ith SNP for the kth ethnic group. We can then define a normalized genotype as


$$G_{\mathrm{ijk}}^{\prime }=\frac{G_{\mathrm{ijk}}-2p_{ik}}{\sqrt{2p_{ik}(1-p_{ik})}},$$

ensuring it has a mean of zero and a variance of one. Under the null hypothesis of no association between the genotypes and the phenotype of interest, the correlation structure of association Z-scores vector, where each Z-score, $Z_{i}$, corresponds to the ith SNP, is expected to mirror that of the original SNP genotypes $G_{i*}$ (Lee *et al.* 2013).

Given that the normalized $G_{i*}^{\prime }$’s are linear combinations of the $G_{i*}$ values, it follows that the SNP Z-scores and the $G_{i*}^{\prime }$ values also share the same correlation structure. Since both have unit variance, they also possess identical covariance structures. Thus, for any s≥1,


$$\text{Cov}(Z_{i},Z_{i+s})=\text{Cov}(G_{i*}^{\prime },G_{i+s*}^{\prime })$$

and this relationship can be expressed as


$$E(Z_{i}\cdot Z_{i+s})=E(G_{i*}^{\prime }\cdot G_{i+s*}^{\prime }).$$

Considering that genotypes from different ethnic groups are independent, we now have:


$$\begin{array}{c} E(Z_{i}\cdot Z_{i+s})=\sum_{k=1}^{K}w_{k}E[{G^{\prime }}_{i*k}\cdot {G^{\prime }}_{i+s*k}]\\ =\sum_{k=1}^{K}w_{k}\text{Cor}({G^{\prime }}_{i*k},{G^{\prime }}_{i+s*k}), \end{array}$$

where $w_{k}$ is the proportion of subjects in the study belonging to the kth ethnic group. Since ${G^{\prime }}_{i*k}$ and ${G^{\prime }}_{i+s*k}$ are values derived from the study cohort genotype, their correlation cannot be directly calculated. However, it can be approximated using equivalent genotype information from the reference panel. Therefore, the mixing proportions, denoted as ${\left[w_{k}\right]}_{N\times 1}$, can be estimated by regressing the product of “reasonably close” SNP Z-scores, $Z_{i}\cdot Z_{i+s}$ against correlation values between standardized genotype vectors at the corresponding SNP pairs for all *N* populations in the reference panel.

Similar to DISTMIX, DISTMIX2 partitions GWAS SNPs into 1000 non-overlapping sets to ensure the SNPs within each set quasi-independent. The mixing proportions ${\left[w_{k}\right]}_{N\times 1}$ are then estimated for each set, with the final weight being computed as the average of ${\left[w_{k}\right]}_{N\times 1}$ across these 1000 sets. However, our previous simulation studies, which explored diverse ancestry composition scenarios, showed that the strategy for SNP selection and estimation is sub-optimal. Association Z-scores provides less information than AFs for estimating ancestry proportions. As a result, a large number of different SNP pairs is required to obtain more reliable results, especially in cases of complex ancestry compositions that include admixed populations such as African or Mexican ancestries in the USA. Another limitation is that the ancestry estimation procedure is integrated within the DISTMIX2 software and cannot be used independently when for estimating ancestry proportions using Z-scores without engaging in the full imputation process.

To address these issues, we developed ZMIX, a refined procedure that implements the process outlined above and incorporates additional steps to improve the accuracy. ZMIX (i) utilizes all possible SNPs pairs in a quasi-independent SNP set to increase the dataset size and (ii) employs quadratic programming for estimating ancestry proportions, thereby boosting accuracy. After determining a quasi-independent SNP set by selecting SNPs such as the 1st, 1001st, 2001st, and so forth, we form all possible SNP pairs within this set (e.g. $Z_{1}\cdot Z_{1001}$, $Z_{1}\cdot Z_{2001}$, etc.). The optimization problem is then formulated as:


$$\underset{w}{\min}\sum_{\overset{i,l}{i\neq l}}{\left(Z_{i}\cdot Z_{l}-\sum_{k=1}^{K}w_{k}\text{Cor}(G_{i*k}^{\prime },G_{l*k}^{\prime })\right)}^{2}$$

subject to the constraints:


$$\sum_{k=1}^{K}w_{k}=1\;\text{and}\;0\leq w_{k}\leq 1,\;k=1,\ldots ,K.$$

We solved this problem using quadratic programming in R, utilizing the “solveQP” function from the quadprog R package (Turlach *et al.* 2019), which essentially amounts to linear regression with preset constraints. The ZMIX method has been implemented as a standalone tool through the “zmix()” function within the GAUSS R package.

### 2.4 Comparison using simulated data

To assess the performance of each method, we conducted extensive simulation studies under various ancestry composition scenarios, as detailed in Algorithm 1. From the simulated genotypes, we calculated reference AFs. Additionally, using the same genotype data and simulated phenotype data, we computed association Z-scores. This step is crucial to enable a direct comparison of ZMIX with RAF-based methods under the identical simulation conditions.

Algorithm 1 Procedure for simulating GWAS summary statistics

1. Define the desired ancestry composition for the simulated GWAS of *N* subjects by specifying proportions $p_{1},p_{2},p_{3},\ldots ,p_{K}$ for each of the *K* ethnic groups in the reference panel.

2. For each ethnic group *k*, determine the number of subjects $n_{k}$ based on the desired composition: $n_{k}=p_{k}\times N$.

3. For each ethnic group *k*, resample $n_{k}$ columns (subjects) with replacement from $G_{k}$, the genotype dataset of the k-th ethnic group in the reference panel, and compile the resampled data into a new matrix $R_{k}$.

4. Combine the columns from all the $R_{k}$ matrices to form the simulated GWAS genotype dataset $G_{\text{sim}}$, ensuring it consists of *N* columns (subjects).

5. Calculate the reference allele frequencies for all variants (rows) in $G_{\text{sim}}$.

6. Generate a vector of *N* standard normal random values to simulate dummy phenotypes. Then, for each SNP (row) in $G_{\text{sim}}$, perform a regression of these phenotypes on the genotypes to derive an association Z-score.

In our simulation study, we considered four ancestry composition scenarios, each with a consistent study size of 10 000 subjects. Table 1 displays the mixing proportions in each scenario as percentages. Cohort 1 consists of 25% CEU, 25% FIN (Finnish in Finland), 25% GBR (British in England and Scotland), and 25% IBS (Iberian Population in Spain). Cohort 2 focuses on the East Asian ethnicity with 25% each from CNE (China North East), CSE (China South East), JPT, and KHV. Cohort 3 features an equal distribution among CCE (China Central East), CEU (Utah residents with Northern and Western European ancestry), and MXL (Mexican ancestry from Los Angeles, USA), with each group comprising 33.33% of the total, representing an even divided composition between ASN (East Asian), EUR (European), and AMR (Admixed American). Cohort 4 represents a more challenging admixed ancestry composition consisting of 50% ACB (African Caribbeans in Barbados), 25% ASW (African Ancestry in Southwest USA), 15% PUR (Puerto Rican in Puerto Rico), and 10% MXL (Mexican Ancestry from Los Angeles, USA), resulting in 75% AFR (African) and 25% AMR (Admixed American). These scenarios were chosen to evaluate of the methods’ performance across diverse ethnic compositions. Each scenario was simulated 100 times and the mean and standard deviation of each method’s mixing proportion estimates were computed for comparison.

**Table 1.** Simulation scenarios.^a^

Scenario	ACB	ASW	CCE	CEU	CNE	CSE	FIN	GBR	IBS	JPT	KHV	MXL	PUR
Cohort 1	0	0	0	25	0	0	25	25	25	0	0	0	0
Cohort 2	0	0	0	0	25	25	0	0	0	25	25	0	0
Cohort 3	0	0	33.3	33.3	0	0	0	0	0	0	0	33.3	0
Cohort 4	50	25	0	0	0	0	0	0	0	0	0	10	15

a Each value indicates the mixing proportion, in terms of percentages, for each population within the four simulated cohorts. These values represent the percentage of the 10 000 simulated subjects that were sampled from each ethnic group.

### 2.5 Comparison using real-world data

We applied DISTMIX, Summix, and ZMIX to three real-world GWAS studies sourced from the Psychiatric Genomic Consortium (PGC). These studies include the 2019 ADHD GWAS (Demontis *et al.* 2019), the 2021 Bipolar Disorder (BP) GWAS (Mullins *et al.* 2021), and the Phase 3 Schizophrenia (SCZ3) GWAS (Trubetskoy *et al.* 2022). The datasets are publicly available at: http://www.med.unc.edu/pgc/. Comparisons based on the real world studies should be approached with caution, as true mixing proportions are rarely explicitly stated and must often be inferred from the corresponding publications. We carefully reviewed each study and estimated the actual ethnic proportions using the information provided. For instance, in the SCZ3 study, specific ancestry proportions of subjects are provided: the SCZ3 GWAS publication (Trubetskoy *et al.* 2022) reports ancestry proportions as 74.3% European (EUR), 17.5% Asian (ASN), 5.7% African American (AA), and 2.5% Latino (LAT).

### 2.6 Application in summary statistics imputation

We assessed the effectiveness of three ancestry estimation methods—DISTMIX, Summix, and ZMIX—by examining the quality of summary statistics imputation achieved using their estimated weights. The imputation process was conducted using DISTMIX’s procedure (Lee *et al.* 2015), which utilizes ancestry proportions to compute ancestry-informed LD. The quality of imputed Z-scores was a key focus, as precise estimation of ethnic mixing proportions is crucial for imputation quality. For this comparison, we focused on the PGC Schizophrenia 3 (Trubetskoy *et al.* 2022) GWAS study. We extracted Illumina Human1M-Duo array SNPs from the study to serve as measured SNPs and used them as input for the imputation process. Initially, we calculated ancestry proportions for each study using the three methods. These calculated weights were then employed in DISTMIX’s imputation procedure to impute Z-scores for the non-Illumina 1M SNPs. Finally, we compared these imputed Z-scores to the reported Z-scores for the same non-Illumina 1M SNPs.

## 3 Results

### 3.1 Simulation

The estimated weights for each cohort, detailed by both population and super population, are presented in Tables 2 and 3. Table 2 focuses solely on those populations used in the simulation studies, providing a detailed view of the estimation accuracy. Cohort 1 was composed of equal proportions (25% each) of CEU, FIN, GBR, and IBS ancestries, aggregating to a total European (EUR) ancestry of 100%. The first row of Tables 2 and 3 shows that both DISTMIX and Summix provided highly accurate estimates of these proportions, achieving 100% EUR estimation. In comparison, ZMIX’s estimation was slightly less accurate, with 94% EUR and small erroneous allocations to African (AFR, 1.5%), admixed American (AMR, 1.8%), East Asian (ASN, 0.9%), and South Asian (SAS, 1.2%) ancestries. DISTMIX2 performed the poorest with 71.5% EUR and significant erroneous allocation to non-European ancestries, e.g. 10.1% AMR. For Cohort 2, characterized by an equal distribution among the CNE, CSE, JPT, and KHV groups (each contributing 25%, totaling 100% ASN), DISTMIX and Summix again accurately captured this composition. ZMIX, while correctly identifying the dominance of ASN ancestry, underestimated it by approximately 3%, erroneously assigning this percentage to non-existent ancestral groups. DISTMIX2 underperformed, correctly estimating only 89% Asian. Cohort 3’s true composition included equal thirds of CCE, CEU, and MXL ancestries (33.33% each), representing an equal distribution across ASN, EUR, and AMR ancestries. In this cohort, as shown in the third row of Tables 2 and 3, both DISTMIX and Summix estimations were highly accurate, with Summix achieving perfect precision. ZMIX performed reasonably well, although it tended to underestimate EUR populations. DISTMIX2 again underperformed, tending to underestimate the AMR and EUR groups, and inaccurately detected large proportions in non-existent AFR and SAS groups. In the more challenging admixed scenario of Cohort 4, consisting of 75% AFR and 25% AMR (i.e. 50% ACB + 25% ASW + 15% PUR + 10% MXL), both allele-frequency-based method, DISTMIX and Summix, performed best. ZMIX captured 76.9% AFR and 17.6% AMR effectively. DISTMIX2 was the least accurate, estimating 68.8% AFR and 12.7% AMR.

**Table 2.** Simulation results by population.^a^

Scenario	Method	ACB	ASW	CCE	CEU	CNE	CSE	FIN	GBR	IBS	JPT	KHV	MXL	PUR
Cohort 1	DISTMIX	0	0	0	21.1	0	0.2	25.2	23.5	24.9	0	0	0	0
	Summix	0	0	0	25	0	0	25	25	25	0	0	0	0
	DISTMIX2	0	0	0	10.1	0	0	21.9	11.6	9.7	0	0	3.5	0
	ZMIX	0	0	0	21.2	0	0	24.9	15.7	18.9	0	0	0	0

	True	0	0	0	25	0	0	25	25	25	0	0	0	0

Cohort 2	DISTMIX	0	0	0	0	25.4	24.2	0	0	0	25	25.1	0	0
	Summix	0	0	0	0	25	25	0	0	0	25	25	0	0
	DISTMIX2	0	0	8.8	0	11.5	22.9	0	0	0	17.0	14.3	0	0
	ZMIX	0	0	6.8	0	16.1	22	0	0	0	23.7	23.3	0	0

	True	0	0	0	0	25	25	0	0	0	25	25	0	0

Cohort 3	DISTMIX	0	0	32.6	29.2	0	0	0	0	0	0	0	33.2	0
	Summix	0	0	33.3	33.3	0	0	0	0	0	0	0	33.3	0
	DISTMIX2	0	0	8.5	0	8.4	5.5	8.4	4.4	0	0	0	12.7	3.4
	ZMIX	0	0	10.7	9.5	7.1	3.6	5.4	3.4	1.5	0	0	30.3	0

	True	0	0	33.3	33.3	0	0	0	0	0	0	0	33.3	0

Cohort 4	DISTMIX	50.1	25	0	0	0	0	0	0	0	0	0	10	14.9
	Summix	50	25	0	0	0	0	0	0	0	0	0	10	15
	DISTMIX2	22.7	17.0	0	0	0	0	1.0	0	1.7	1.2	0	2.6	4.5
	ZMIX	34.6	31.0	0	0	0	0	0	0	0	0	0	6.6	8.8

	True	50	25	0	0	0	0	0	0	0	0	0	10	15

a Estimated ancestry proportions from all four methods across Cohorts 1, 2, 3, and 4 are presented. The actual ancestry proportions from which the study was simulated are indicated in the rows marked “True.” All values are presented as percentages. For brevity, only populations used in simulations are shown.

**Table 3.** Simulation results by super population.^a^

Scenario	Method	AFR	AMR	ASN	EUR	SAS
Cohort 1	DISTMIX	0	0	0	100	0
	Summix	0	0	0	100	0
	DISTMIX2	5.5	10.1	4.9	71.5	8.0
	ZMIX	1.5	1.8	0.9	94.7	1.2

	True	0	0	0	100	0

Cohort 2	DISTMIX	0	0	100.6	0	0
	Summix	0	0	100	0	0
	DISTMIX2	4.1	2.4	89.0	1.3	3.3
	ZMIX	2.4	0.2	97	0.2	0.3

	True	0	0	100	0	0

Cohort 3	DISTMIX	0	33.3	33.5	34.9	0.1
	Summix	0	33.3	33.3	33.3	0
	DISTMIX2	7.0	25.3	33.7	23.0	11.1
	ZMIX	4.4	33.9	32.8	26.3	2.7

	True	0	33.3	33.3	33.3	0

Cohort 4	DISTMIX	75.1	24.9	0	0	0
	Summix	75	25	0	0	0
	DISTMIX2	68.8	12.7	5.5	5.5	7.5
	ZMIX	76.9	17.6	1.5	2.9	1.1

	True	75	25	0	0	0

a Individual populations detailed in Table 4 were grouped by their respective super populations, and their weights were aggregated accordingly.

AFR = African; AMR = Admixed American; ASN = East Asian; EUR = European; SAS = South Asian.

Overall, while DISTMIX and Summix consistently provided highly accurate estimations across all four simulated cohorts, ZMIX exhibited a marginally lower performance when compared to these AF-based methods. Nonetheless, considering that ZMIX operates on less informative association Z-scores, its comparative performance is notably robust and demonstrates potential utility in scenarios where AF data are not available. As expected, DISTMIX2 had the weakest performance in all four scenarios. This highlights that the enhancements in DISTMIX2 (i.e. ZMIX), including a better SNP selection procedure and the use of quadratic programming, significantly improve its performance.

### 3.2 Real-world studies

The top performers in simulation studies—DISTMIX, Summix, and ZMIX—were applied to three PGC GWAS studies: the Phase 1 ADHD (Demontis *et al.* 2019), the Phase 2 Bipolar Disorder (Mullins *et al.* 2021), and Phase 3 Schizophrenia (Trubetskoy *et al.* 2022). Determining the exact ethnic mixing proportions in these studies is challenging, but rough estimates can be gleaned from the papers associated with each study. Our comparison primarily focuses on the estimated super populations due to limited information on the individual populations involved. The estimated weights for each study, broken down by population and super population, are presented as percentages in Tables 4 and 5.

**Table 4.** PGC study results by population.^a^

Study	Method	CCE	CCS	CDX	CEU	CNE	CSE	FIN	GBR	JPT	KHV	IBS	ORK	TSI
ADHD	DISTMIX	0	0	0	65.7	0	1.6	1.8	7.8	0	0	0	21.6	0
	Summix	0	0	0	62.2	0	0	3.0	8.1	0	0	0	26.7	0
	ZMIX	0	0	1.7	32.9	0	0	12.2	15.9	0	0	10.5	18.7	3.1

BP	DISTMIX	0	0	0	57.0	4.1	0	3.6	7.2	0	0	0	25.2	0
	Summix	0	0	0	50.4	0	0	6.4	7.0	0	0	0	36.0	0
	ZMIX	0	0	0	45.4	0	2.6	13.8	8.6	0	0	5.1	20.9	0

SCZ3	DISTMIX	4.0	3.0	3.9	31.7	3.7	0	4.7	5.6	4.1	5.0	7.0	23.2	2.2
	Summix	0	1.3	4.1	35.2	3.6	0	5.2	6.3	4.8	3.3	7.8	24.8	2.1
	ZMIX	0	0	6.3	64.7	0	5.9	3.9	0	0.2	0	0	0	4.2

a In the study column, “ADHD” refers to the PGC ADHD GWAS, “BP” to the PGC Phase 2 Bipolar GWAS, and “SCZ3” to the PGC Phase 3 Schizophrenia study. For brevity, only populations with estimated weights >1% are shown.

**Table 5.** PGC study results by super population.^a^

Study	Method	AFR	AMR	ASN	EUR	SAS
ADHD	DISTMIX	0.7	0.3	1.7	96.9	0.5
	Summix	0	0	0	100	0
	ZMIX	0	0.2	2.1	93.3	4.4

BP	DISTMIX	1.6	0.6	4.6	92.9	0.3
	Summix	0	0.2	0	99.8	0
	ZMIX	2.0	0.7	3.0	93.7	0.6

SZ3	DISTMIX	0.8	0.8	23.5	74.3	0.6
	Summix	0.6	0.9	17.1	81.4	0
	ZMIX	4.0	4.4	12.4	72.9	6.4

a Weights for the individual 29 populations were grouped together by their super population and aggregated. The weights are presented as percentages.

AFR = African; AMR = Admixed American; ASN = East Asian; EUR = European; SAS = South Asian.

According to the Supplementary Materials for the PGC ADHD (2019) study (Demontis *et al.* 2019), approximately 5% of the subjects were of Asian ancestry, 3% were from the USA with “diverse” ancestry, and the remainder had European ancestry. Based on their descriptions, we approximate that the study comprises roughly 92% European and 5% Asian, and a small segment (3%) of Admixed American ancestry. The estimated weights by individual population and super population are presented in the first row of Tables 4 and 5, respectively. In this study, DISTMIX and ZMIX provided more accurate estimations as they both identified the presence of a small non-European population. While Summix estimated the study to be entirely European, which is partially accurate, it overlooks the non-European segments. Notably, ZMIX more effectively detected the Asian populations, estimating a combined 6.5% between East Asian and South Asian populations.

The PGC Bipolar Phase 2 study (Mullins *et al.* 2021) reports that the participants are primarily from European, North American, and Australian backgrounds. Notably, this cohort may include some African subjects, as the ASW (African Ancestry in Southwest USA) is one of the African sub-populations. Geographically, Australia is proximate to many Southern and Eastern Asian countries, which might explain the presence of Asian populations. This is consistent with the estimates from DISTMIX and ZMIX, which both detect about 1%–2% of African and a total of 4%–5% of Asian populations, respectively. In contrast, Summix estimates that the entire study comprises predominantly European subjects.

The PGC Schizophrenia Phase 3 study (Trubetskoy *et al.* 2022) reports the ethnic proportions as approximately 74.5% European, 17.5% East Asian, 5.7% African, and 2.5% Latino. In terms of estimating the European proportion, DISTMIX and ZMIX perform better than Summix, although DISTMIX tends to overestimate, and ZMIX underestimates the East Asian population. Summix provides the most accurate estimate for the East Asian group but slightly overestimates the European segment. ZMIX significantly overestimates the African and Latino populations. However, considering the potential relatedness between South Asian and Eastern Asian populations, ZMIX does a commendable job in estimating the total Asian and European populations.

While the exact ethnic composition of the PGC Bipolar Phase 2 study is not fully detailed, DISTMIX appears to provide the most accurate estimations among the three methods. Summix accurately estimates the East Asian population for the Schizophrenia study, but it calculates a 0% representation for both the ADHD and Bipolar studies, which is known to be incorrect, at least for the ADHD study. ZMIX delivers results very similar to DISTMIX for all studies, except for some discrepancies in the Schizophrenia GWAS. This is particularly noteworthy considering that more recent GWAS often do not publicly disclose AFs.

### 3.3 Imputation

Correlation plots illustrating the relationship between Z-scores imputed by DISTMIX’s imputation procedure and the reported Z-scores from the PGC Schizophrenia 3 study are included in the Supplementary Fig. S1. The squared correlation coefficients between Z-scores exceeded 0.99 for all cases, regardless of the weight estimation method employed to estimate ancestry-informed LD in DISTMIX’s imputation procedure. Furthermore, the imputation quality of Z-scores imputed by DISTMIX is represented through a color gradient, predominantly indicating an imputation information range of 0.9–1. This demonstrates that the three weight estimation methods—DISTMIX, Summix, and ZMIX—consistently resulted in high-quality imputations, on par with those produced by IMPUTE2.

### 3.4 Ease of use

Both DISTMIX and ZMIX are more user-friendly compared to Summix, as they only require file paths for an input text file containing GWAS summary statistics and for component files of the reference panel. The input text file should include columns for rsid (SNP ID), chromosome number, base-pair location, allele 1 (reference allele), allele 2 (alternative allele), and either reference AF for DISTMIX or Z-score for ZMIX. The “afmix()” function, implementing the DISTMIX’s ancestry proportion estimation method, and the “zmix()” function, implementing the ZMIX method, are both developed in Rcpp/C++ for efficient computation and are available within the GAUSS R package (https://github.com/statsleelab/gauss). The 1000 Genomes panel and the more extensive 33KG panel are readily available to users and are seamlessly integrated with the GAUSS R package, eliminating the need for users to obtain reference AF or genotype information from a reference panel separately.

In contrast, Summix requires a data.frame object that includes columns for basic items such as rsid, chromosome number, etc., along with individual RAFs from the reference panel for each population under consideration. To use Summix, users need to extract individual RAFs from a reference panel and provide this information to Summix for analysis. Given that many reference panels consist of millions of SNPs, this process introduces significant computation time. Data comprising several million rows and over 20 columns (as in the case with all 29 populations in the 33KG panel) must be loaded into R before computation can begin.

## 4 Conclusions

In this article, we introduce ZMIX, a novel method for estimating ancestry proportions using only GWAS association Z-scores. ZMIX builds upon the ancestry estimation procedure implemented in DISTMIX2 (Chatzinakos *et al.* 2021), incorporating modifications that significantly enhance the accuracy. These improvements include refined SNP pair selection procedures and the use of quadratic programming for solving equations more effectively. We assessed ZMIX’s performance against its predecessor (DISTMIX2) and two established RAF-based ancestry proportion estimation methods (DISTMIX and Summix) through extensive simulation studies under various ancestry composition scenarios. Additionally, we applied ZMIX, DISTMIX, and Summix to real-world GWAS data from the PGC ADHD Phase 1 Study (Demontis *et al.* 2019), the PGC Phase 2 Bipolar Study (Mullins *et al.* 2021), and the PGC Phase 3 Schizophrenia Study (Trubetskoy *et al.* 2022). Estimated ancestry proportions were then utilized in summary statistics imputation procedures to determine which method yields the highest quality imputed Z-scores. This approaches enable us to compare the efficacy of each method in actual research settings.

In simulation studies, ZMIX did not perform as well in estimating proportions across individual populations compared to DISTMIX and Summix. However, at the super population level, ZMIX demonstrated comparable performance to, or slightly underperformed, the two allele-frequency-based methods and significantly outperformed DISTMIX2. It provided reasonable estimates for all four simulated cohorts at the super population level, though its predictions for the ethnically more diverse and admixed Cohorts 3 and 4 were somewhat less precise. This issue can be partly attributed to the zero-inflated reference allele counts for certain populations in the 33KG reference panel, which complicated the calculation of simulated Z-scores for specific cohorts. Unlike ZMIX, DISTMIX, and Summix were not significantly impacted by these extreme RAF values.

In our comparative analysis using PGC data, ZMIX showed notable performance relative to DISTMIX and Summix. While ZMIX did overestimate certain populations, particularly the South Asian proportions in the ADHD and Schizophrenia studies, its overall estimates aligned well with those from DISTMIX. Conversely, Summix had a tendency to overestimate European populations and, with the exception of East Asia in the Schizophrenia study, typically assigned zero weights to all non-European populations. The imputation results for ZMIX were promising, as it delivered high-quality imputations even while slightly overestimating the mixing proportions for certain populations. Most imputation information values generated were high, comparable to those achieved by established methods. This similarity in results is especially noteworthy, given that ZMIX relies solely on association Z-scores, which generally contain less ancestral composition information than AF data.

Despite its promising results, ZMIX has limitations when compared to RAF-based methods. A significant constraint is ZMIX’s reliance on summary statistics from SNPs across all chromosomes to accurately estimate ancestry proportions. In contrast, RAF-based methods can produce highly accurate estimates using summary data from just a single chromosome or even specific genomic regions spanning several megabases. This requirement of ZMIX for comprehensive chromosomal data could limit its applicability in scenarios with restricted genetic information. One potential approach to enhance ZMIX’s performance with fewer SNPs is the utilization of ancestry informative SNPs. By identifying and using Z-scores from these specific SNPs, ZMIX could more effectively infer ancestry proportions, even with a reduced genomic dataset. Another possible limitation arises from the simplicity of our Z-score simulation method. Although many publicly available GWAS summary statistics are adjusted for ancestry components, we simulated Z-scores from models not adjusted for ancestry and used them in ZMIX analyses. ZMIX operates under the assumption that the correlation between SNP Z-scores reflects the correlation between SNP genotypes of the study cohorts. We believe that this assumption holds true even when models are adjusted for ancestry, as these adjustments tend to primarily affect the magnitude of the SNP effects, not their correlation structure. This is supported by real data analyses using PGC GWAS data, which are adjusted for ancestry, where ZMIX performed quite effectively. Nonetheless, investigating the performance of ZMIX with Z-scores derived from ancestry-adjusted models could offer intriguing insights and warrants further exploration.

In conclusion, we successfully developed ZMIX, an accurate ancestry estimation method using GWAS association Z-scores. This method consistently yielded results comparable to the two allele-frequency-based methods, DISTMIX and Summix, in both simulation studies and real-world GWAS studies. In certain cases, it even surpassed the performance of the allele-frequency-based methods. The significance of these results lies in its potential to enable researchers to accurately account for complex ancestry composition in large multi-ethnic GWAS, particularly in situations where allele-frequencies are not publicly accessible.

## Supplementary Material

vbae128_Supplementary_Data

## Data Availability

The current implementation of the ZMIX function is available in the “gauss” R package, hosted in the GitHub repository at https://github.com/statsleelab/gauss. The 33KG reference panel data can be downloaded from https://statsleelab.github.io/gauss/articles/ref_33KG.html.

## References

[vbae128-B1] Arriaga-MacKenzie IS , Matesi G, Chen S et al Summix: a method for detecting and adjusting for population structure in genetic summary data. Am J Hum Genet 2021;108:1270–82. 34157305 10.1016/j.ajhg.2021.05.016 PMC8322937

[vbae128-B2] Byrska-Bishop M , Evani US, Zhao X et al; Human Genome Structural Variation Consortium. High-coverage whole-genome sequencing of the expanded 1000 genomes project cohort including 602 trios. Cell 2022;185:3426–40.e19. 36055201 10.1016/j.cell.2022.08.004 PMC9439720

[vbae128-B3] Cai N , Bigdeli T, Kretzschmar W et al 11,670 whole-genome sequences representative of the Han Chinese population from the converge project. Sci Data 2017;4:170011. 28195579 10.1038/sdata.2017.11 PMC5308202

[vbae128-B4] Chatzinakos C , Lee D, Cai N et al Increasing the resolution and precision of psychiatric genome-wide association studies by re-imputing summary statistics using a large, diverse reference panel. Am J Med Genet B Neuropsychiatr Genet 2021;186:16–27. 33576176 10.1002/ajmg.b.32834 PMC8247874

[vbae128-B5] Demontis D , Walters RK, Martin J et al; 23andMe Research Team. Discovery of the first genome-wide significant risk loci for attention deficit/hyperactivity disorder. Nat Genet 2019;51:63–75. 30478444 10.1038/s41588-018-0269-7 PMC6481311

[vbae128-B6] Freedman ML , Reich D, Penney KL et al Assessing the impact of population stratification on genetic association studies. Nat Genet 2004;36:388–93. 15052270 10.1038/ng1333

[vbae128-B7] Guo MH , Plummer L, Chan YM et al Burden testing of rare variants identified through exome sequencing via publicly available control data. Am J Hum Genet 2018;103:522–34. 30269813 10.1016/j.ajhg.2018.08.016 PMC6174288

[vbae128-B8] Hendricks AE , Billups SC, Pike HNC et al ProxECAT: proxy external controls association test. A new case-control gene region association test using allele frequencies from public controls. PLoS Genet 2018;14:e1007591. 30325923 10.1371/journal.pgen.1007591 PMC6191077

[vbae128-B9] Homer N , Szelinger S, Redman M et al Resolving individuals contributing trace amounts of DNA to highly complex mixtures using high-density SNP genotyping microarrays. PLoS Genet 2008;4:e1000167. 18769715 10.1371/journal.pgen.1000167 PMC2516199

[vbae128-B10] Lee D , Bacanu S-A. GAUSS: a summary-statistics-based R package for accurate estimation of linkage disequilibrium for variants, Gaussian imputation, and TWAS analysis of cosmopolitan cohorts. Bioinformatics 2024;40:btae203. 38632050 10.1093/bioinformatics/btae203 PMC11052653

[vbae128-B11] Lee D , Bigdeli TB, Riley BP et al DIST: direct imputation of summary statistics for unmeasured SNPs. Bioinformatics 2013;29:2925–7. 23990413 10.1093/bioinformatics/btt500 PMC3810851

[vbae128-B12] Lee D , Bigdeli TB, Williamson VS et al DISTMIX: direct imputation of summary statistics for unmeasured SNPs from mixed ethnicity cohorts. Bioinformatics 2015;31:3099–104. 26059716 10.1093/bioinformatics/btv348 PMC4576696

[vbae128-B13] McCarthy S , Das S, Kretzschmar W et al; and the Haplotype Reference Consortium. A reference panel of 64,976 haplotypes for genotype imputation. Nat Genet 2016;48:1279–83. 27548312 10.1038/ng.3643 PMC5388176

[vbae128-B14] Mullins N , Forstner AJ, O'Connell KS et al; HUNT All-In Psychiatry. Genome-wide association study of more than 40,000 bipolar disorder cases provides new insights into the underlying biology. Nat Genet 2021;53:817–29. 34002096 10.1038/s41588-021-00857-4 PMC8192451

[vbae128-B15] Need AC , Goldstein DB. Next generation disparities in human genomics: concerns and remedies. Trends Genet 2009;25:489–94. 19836853 10.1016/j.tig.2009.09.012

[vbae128-B16] Popejoy AB , Fullerton SM. Genomics is failing on diversity. Nature 2016;538:161–4. 27734877 10.1038/538161a PMC5089703

[vbae128-B17] Price AL , Patterson NJ, Plenge RM et al Principal components analysis corrects for stratification in genome-wide association studies. Nat Genet 2006;38:904–9. 16862161 10.1038/ng1847

[vbae128-B18] Trubetskoy V , Pardiñas AF, Qi T et al; Schizophrenia Working Group of the Psychiatric Genomics Consortium. Mapping genomic loci implicates genes and synaptic biology in schizophrenia. Nature 2022;604:502–8. 35396580 10.1038/s41586-022-04434-5 PMC9392466

[vbae128-B19] Turlach BA , Weingessel A, Moler C. quadprog: functions to solve quadratic programming problems. R Package Version 1.5-8. 2019. doi: 10.32614/CRAN.package.quadprog.

[vbae128-B20] Uhlerop C , Slavković A, Fienberg SE. Privacy-preserving data sharing for genome-wide association studies. J Priv Confid 2013;5:137–66. 26525346 PMC4623434

[vbae128-B21] Zhang J , Stram DO. The role of local ancestry adjustment in association studies using admixed populations. Genet Epidemiol 2014;38:502–15. 25043967 10.1002/gepi.21835 PMC5079159

