# Supplementary material for: ZMIX: estimating ancestry proportions using GWAS association Z-scores
Source: Bioinform Adv. 2024 Aug 29;4(1):vbae128. doi: 10.1093/bioadv/vbae128 (PMC11632184; doi:10.1093/bioadv/vbae128)

ZMIX: Estimating ancestry proportions using GWAS association Z-scores

Supplementary Data

Trent Dennis[1,2] and Donghyung Lee[1,*]

[1]Department of Statistics, Miami University, 105 Tallawanda Rd., Oxford, Ohio, 45056, USA
[2]Winton Hill Business Center, P&G, 6300 Center Hill Ave, Cincinnati, Ohio, 45232, USA

*Correspondence: leed13@miamioh.edu

Supplementary Table 1. The population group, the number of subjects, corresponding super population, and description for all 29 population groups in the 33KG reference panel. AFR: African, AMR: Admixed American, ASN: East Asian, EUR: European, SAS: South Asian

Population	Sample Size	Super Population	Description
ACB	164	AFR	African Caribbeans in Barbados
ASW	162	AFR	African Ancestry in Southwest US
BEB	86	SAS	Bengali from Bangladesh
CCE	3409	ASN	China Central East
CCS	2613	ASN	China Central South
CDX	95	ASN	Chinese Dai in Xishuangbanna, China
CEU	6360	EUR	Utah residents with Northern and Western European ancestry
CLM	98	AMR	Colombians from Medellin, Colombia
CNE	2330	ASN	China North East
CSE	2020	ASN	China South-East
ESN	140	AFR	Esan in Nigeria
FIN	3529	EUR	Finnish in Finland
GBR	2020	EUR	British in England and Scotland
GIH	110	SAS	Gujarati Indian from Houston, Texas
GWD	113	AFR	Gambian in Western Divisions in the Gambia
IBS	1309	EUR	Iberian Population in Spain
ITU	95	SAS	Indian Telugu from the UK
JPT	107	ASN	Japanese in Tokyo, Japan
KHV	226	ASN	Kinh in Ho Chi Minh City, Vietnam
LWK	99	AFR	Luhya in Webuye, Kenya
MSL	87	AFR	Mende in Sierra Leone
MXL	187	AMR	Mexican Ancestry from Los Angeles, USA
ORK	5772	EUR	Orkney Island study
PEL	110	AMR	Peruvians from Lima, Peru
PJL	121	SAS	Punjabi from Lahore, Pakistan
PUR	138	AMR	Puerto Rican in Puerto Rico
STU	110	SAS	Sri Lankan Tamil from the UK
TSI	1291	EUR	Toscani in Italia
YRI	52	AFR	Yoruba in Ibadan, Nigeria

Supplementary Figure 1. Correlation plots comparing Z-scores imputed by DISTMIX with the reported Z-scores from the PGC Schizophrenia 3 study. Ancestry proportions estimated by Summix, DISTMIX, and ZMIX were input into DISTMIX's imputation procedure to estimate ancestry-informed linkage disequilibrium (LD) and perform summary statistics imputation. The squared correlation coefficient, rounded to three decimal places, is displayed above each plot. Additionally, the imputation information for each Z-score, as determined by DISTMIX's imputation process, is quantified using the color scale in the legend on the right-hand side.

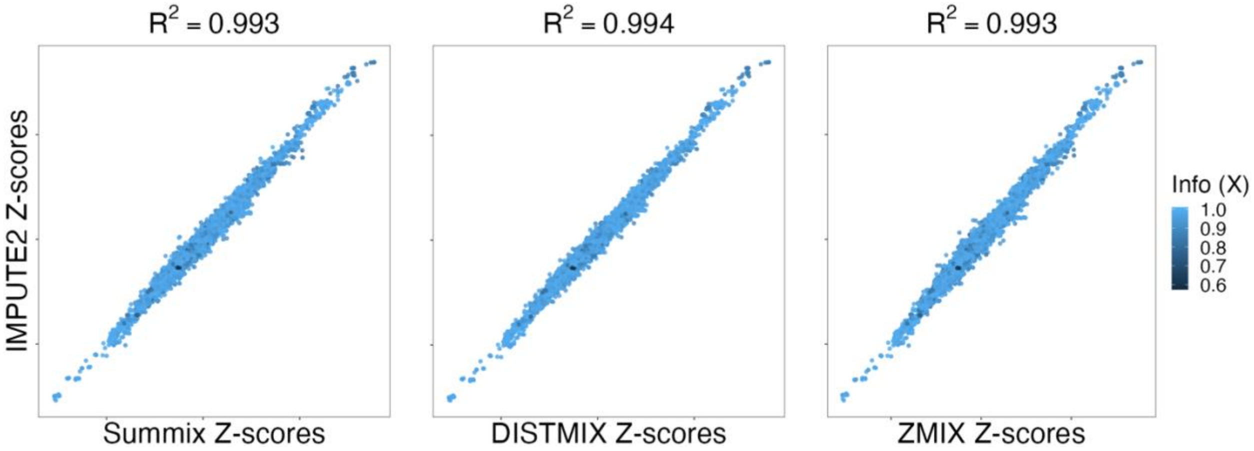

Supplement: vbae128_Supplementary_Data [file vbae128_supplementary_data.pdf]
